# HiCRep.py: fast comparison of Hi-C contact matrices in Python

**DOI:** 10.1093/bioinformatics/btab097

**Published:** 2021-02-12

**Authors:** Dejun Lin, Justin Sanders, William Stafford Noble

**Affiliations:** Department of Genome Sciences, University of Washington, Seattle, WA 98040, USA; Department of Computer Science, Brown University, Providence, RI 02912, USA; Department of Genome Sciences, University of Washington, Seattle, WA 98040, USA; Paul G. Allen School of Computer Science and Engineering, University of Washington, Seattle, WA 98040, USA

## Abstract

**Motivation:**

Hi-C is the most widely used assay for investigating genome-wide 3D organization of chromatin. When working with Hi-C data, it is often useful to calculate the similarity between contact matrices in order to assess experimental reproducibility or to quantify relationships among Hi-C data from related samples. The HiCRep algorithm has been widely adopted for this task, but the existing R implementation suffers from run time limitations on high-resolution Hi-C data or on large single-cell Hi-C datasets.

**Results:**

We introduce a Python implementation of HiCRep and demonstrate that it is much faster and consumes much less memory than the existing R implementation. Furthermore, we give examples of HiCRep’s ability to accurately distinguish replicates from non-replicates and to reveal cell type structure among collections of Hi-C data.

**Availability and implementation:**

HiCRep.py and its documentation are available with a GPL license at https://github.com/Noble-Lab/hicrep. The software may be installed automatically using the pip package installer.

**Supplementary information:**

Supplementary data are available at *Bioinformatics* online.

## 1 Introduction

Hi-C is a powerful genomic assay for quantifying chromatin interactions across the whole genome (Lieberman-Aiden *et al.*, 2009). It has been used extensively to study genome architecture and function in many different species and to understand how genome structure affects genetic diseases. The result of a Hi-C experiment is typically processed into a matrix, whose entries are contact counts between pairs of genomic loci. As Hi-C experiments become more popular, tools that are able to efficiently perform analysis on the resulting contact matrices are in increasing demand (Ay and Noble, 2015).

A common task in Hi-C data analysis is measuring the similarity between pairs of datasets. One application of Hi-C similarity is to assess experimental reproducibility. Low reproducibility may indicate low experiment quality or low sequencing depth. Low reproducibility may also warn against merging multiple Hi-C replicates, which is a common practice to boost the signal-to-noise ratio (Yardımcı *et al.*, 2019). HiCRep is a tool for quantifying the similarity between pairs of Hi-C contact matrices based on their stratum-adjusted correlation coefficients (SCCs) (Yang *et al.*, 2017). The SCC is a correlation score ranging from –1 and 1, where a higher score suggests higher similarity between the two input Hi-C matrices. Using high SCC scores as a proxy for high reproducibility, a number of published works have used HiCRep to assess the quality of replicate experiments and to make sure that merging them is sound (Li *et al.*, 2020; Pal *et al.*, 2019) or to validate that data from a novel assay closely resembles traditional Hi-C data (Lee *et al.*, 2019; Li *et al.*, 2019). Beyond comparing replicates, HiCRep has also proved useful as a tool for measuring quantitative differences among samples. For example, Ray *et al.* (2019) used HiCRep to compare Hi-C contact maps of samples before and after undergoing heat shock in order to determine whether the shock had an effect on chromatin structure. HiCRep can also be used to help interpret single-cell Hi-C (scHi-C) data. For example, Liu *et al.* (2018) demonstrated that the SCC values calculated by HiCRep can be used as the basis for a multidimensional scaling (MDS) visualization that accurately captures cell cycle structure in scHi-C data.

The original implementation of HiCRep was released as an R package (Yang *et al.*, 2017). One of its the biggest drawbacks is its inefficiency, mainly because of the dependence on dense contact matrix operations. In a head-to-head comparison against three other tools for measuring reproducibility, HiCRep was found to be the slowest by a significant margin (Yardımcı *et al.*, 2019). This means that applying the R implementation to Hi-C data at high resolution or to large scHi-C datasets is prohibitively slow.

Here we present a Python implementation of the HiCRep algorithm that is much faster than its predecessor. Our Python version implements all operations using sparse matrices, which greatly reduce the memory consumption and computation time. Additionally, we have made the software more accessible by providing a command line interface as well as a Python application programming interface. 

## 2 Implementation

HiCRep takes as input two Hi-C contact matrices in either .cool or .mcool format (Abdennur and Mirny, 2019). First, matrices are normalized by the total contact counts and smoothed with a 2D mean filter of size set by the user. Then, corresponding diagonals of the two contact matrices are compared and used to calculate SCC scores, as described in the original HiCRep paper (Yang *et al.*, 2017). The software produces as output a list of SCC scores per chromosome. This output faithfully matches that produced by the existing R implementation (see Supplementary Material for details). We provide thorough unit tests of the implementation covering most of its functionality.

## 3 Results

**Fig. 1.**
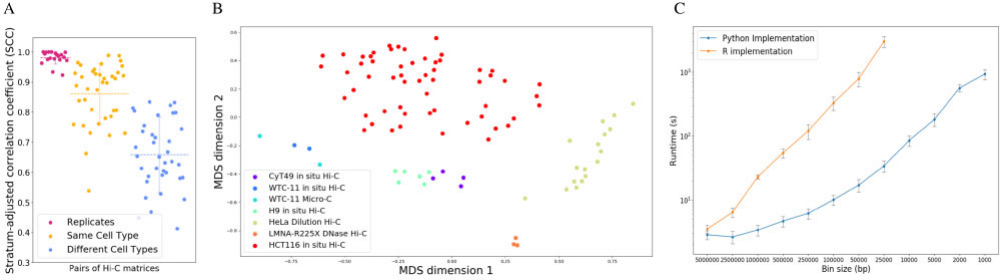
(**A**) The figure plots the HiCRep score for various pairs of Hi-C experiments, including biological replicates (red), non-replicate experiments of the same cell type (green) and non-replicate experiments of different cell types (blue). Horizontal lines with error bars correspond to the mean and SD of each group. (**B**) MDS plot based on HiCRep scores from 90 Hi-C experiments carried out on a variety of cell types. (**C**) Timing comparison of the R and Python implementations of HiCRep for Hi-C matrices with varying bin sizes. Error bars are standard deviation over five runs. Run times for the R implementation beyond 10 kb resolution are not shown, as the program required more memory for these calculations than was available (see Supplementary Fig. S1)

We used HiCRep to calculate SCC scores between 95 pairs of publicly available Hi-C matrices—19 pairs of biological replicates, 38 pairs of non-replicates of the same cell type and 38 pairs of non-replicates of different cell types. As shown in Figure 1A, pairs of replicates consistently exhibit very high SCC scores (mean: 0.98, SD: 0.02), which are markedly higher than the scores of both non-replicates of the same cell type (mean: 0.86, SD: 0.10) and non-replicates of different cell types (mean: 0.61, SD: 0.16). These results suggest that HiCRep does a good job of capturing the reproducibility of Hi-C datasets and is able to accurately separate replicates from non-replicates.

We also used HiCRep to evaluate the pairwise SCC scores of 90 Hi-C experiments conducted by the 4D Nucleome Consortium on a number of different cell types (Supplementary Table S1). Using these SCC scores as the distance metric for an MDS model, we show that HiCRep reveals structure among the experiments, with different cell types clustering separately (Fig. 1C).

Finally, we compared the run times of our implementation of HiCRep to the R implementation. We selected five pairs of high-resolution Hi-C experiments and ran both implementations of HiCRep on each of them at a number of different resolutions (Fig. 1B). Comparing the runtimes, we see that at higher resolutions the Python implementation of HiCRep is more than 20 times faster than the R version. This speed increase allows our version of HiCRep to be practically applied to data with much smaller bin sizes or to larger collections of scHi-C data than was previously possible.

## Funding

This work has been supported by the National Institutes of Health awards [grant numbers U54 DK107979, UM1 HG011531].

*Conflict of Interest*: none declared.

## Supplementary Material

btab097_Supplementary_Data
