# Supplementary material for: HiCRep.py: fast comparison of Hi-C contact matrices in Python
Source: Bioinformatics. 2021 Feb 12;37(18):2996–7. doi: 10.1093/bioinformatics/btab097 (PMC8479650; doi:10.1093/bioinformatics/btab097)
Supplement: btab097_Supplementary_Data [file btab097_supplementary_data.zip › hicrep_supplement.pdf]

Supplement

Verifying the reimplementation In order to ensure that our Python implementation reproduces the results of the R version, we ran both versions on five pairs of Hi-C data sets with various parameter settings and resolutions ranging from 25 kb to 5 Mb, and verified that our SCC scores matched those produced by the R implementation (version 1.11.0) to three decimal places. We also include in our implementation a comprehensive set of unit tests that ensure critical functions are working as expected.

Memory To compare the memory usage of the two implementations, we used the same five pairs of high resolution Hi-C experiments we used for the timing experiment and plotted the peak memory usage of both implementations (Supplementary Figure 1). At the highest resolution we were able to run (10 kb), the R implementation of HiCRep uses 16 times as much memory as our Python version.

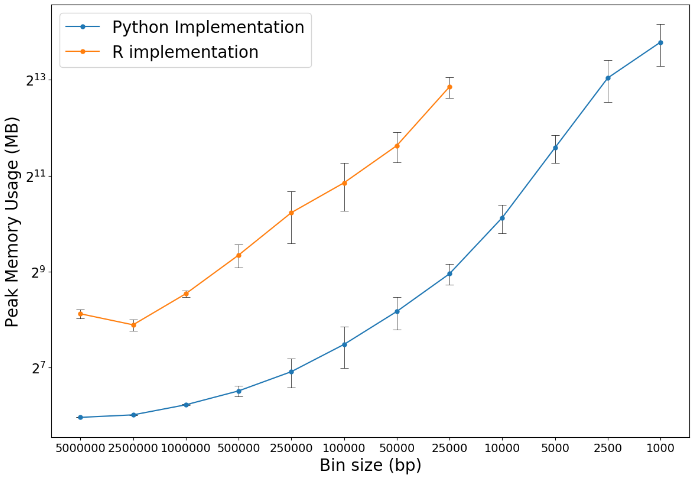

Figure 1: Comparison of peak memory consumptions of the R and Python implementations of HiCRep for Hi-C matrices with varying bin sizes. Error bars are standard deviation over five runs. Values for the R implementation at bin sizes 10 kb and below are not shown, as the memory and runtime requirements of the program at these resolutions made running it intractable.

Comparison to existing Python implementation During review of this manuscript, we were made aware of another Python implementation of HiCRep (https://github.com/cmdoret/hicreppy), which also makes use of sparse matrices. Comparing this existing python implementation of HiCRep to our own, we find that our version runs much faster (Supplementary figure 2A) and consumes less memory (Supplementary figure 2B). Most notably, we observe an exponential speedup of our version over the existing python implementation in bin sizes less than 100 kb (Supplementary figure 2C).

Furthermore, our implementation offers an API in addition to the command line interface and is more faithful to the original HiCRep implementation by reporting SCC scores for each chromosome rather than a single combined SCC score for the genome as a whole. However, the existing implementation includes the functionality of finding the optimal value for the smoothing parameter at a given resolution, which we omitted in favor of having the user use the values identified by the authors of the original HiCRep implementation.

Data All of the Hi-C contact matrices presented here were obtained from the 4DN data portal, where they are available in Cooler format (`https://data.4dnucleome.org`). A list of accession numbers for the files we used are in Supplementary Table 1. For our analyses we used these data sets at a resolution of 500 kb, and we performed no normalization on any of the contact matrices.

Comparison of replicates To assess how well HiCRep can separate replicates from non-replicates, we downloaded 19 pairs of replicates from 4DN. To produce Figure 1A, we used HiCRep to compute SCC

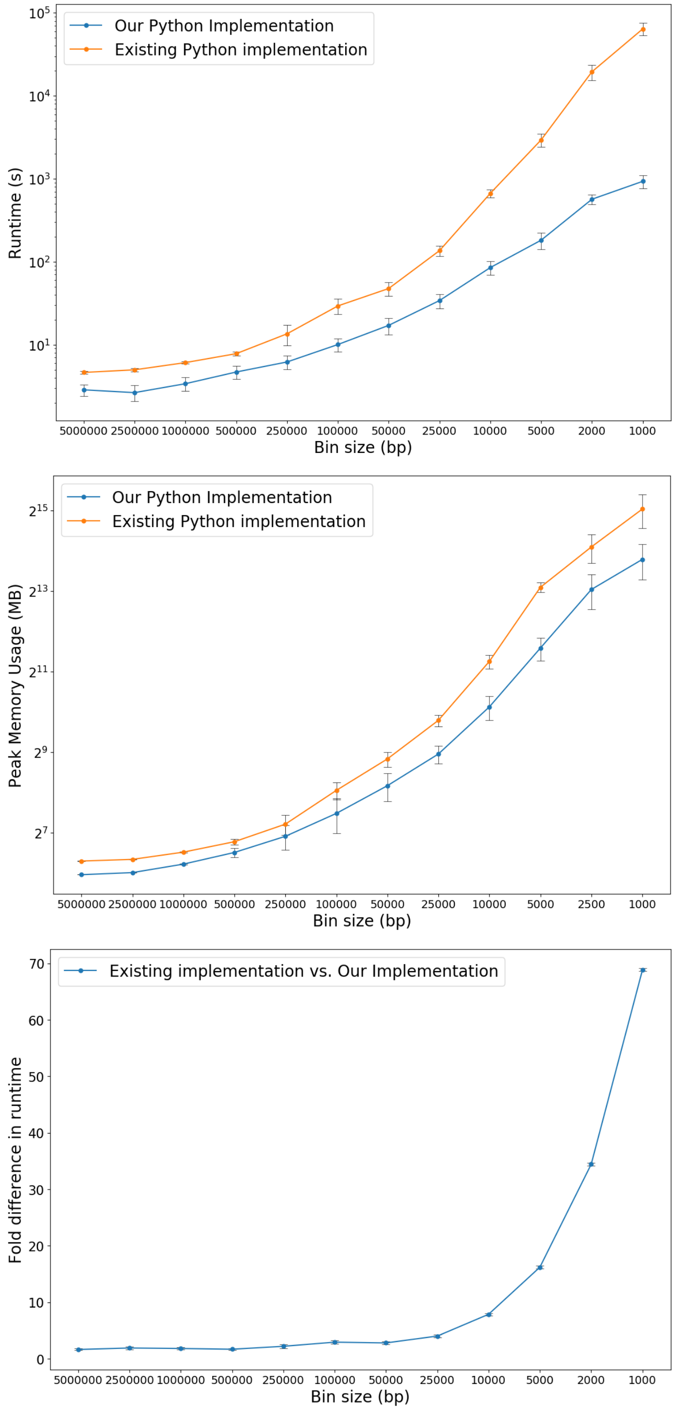

A

B

C

Figure 2: **(A)** Timing comparison of our implementation to the existing Python implementation of HiCRep for Hi-C matrices with varying bin sizes. Error bars are standard deviation over five runs. **(B)** Comparison of peak memory consumption of our implementation to the existing Python implementation of HiCRep for the same Hi-C matrices as above. Error bars are also standard deviation over five runs. **(C)** The magnitude of the speedup achieved by our implementation, plotted as the fold difference between the two run times shown in figure A.

scores for the 19 replicate pairs as well as 38 randomly selected pairs of data sets from the same "biosource" (i.e., cell type or tissue sample) and 38 randomly selected pairs from different biosources. A list specifying specifically which pairs of the data sets were used during this analysis is provided in Supplementary Table 2. For this experiment, HiCRep was run with a bin size of 500 kb, a smoothing factor $h = 2$, a maximum genomic distance of 5 Mb, and down-sampling set to false.

Multidimensional scaling The MDS plot in Figure 1B was produced by using HiCRep to obtain pairwise SCC scores for 90 Hi-C datasets where replicates had already been merged. We then converted these correlation coefficients to Euclidean distance with the equation $d(A, B) = \sqrt{1 - SCC(A, B)}$. Finally, these distances were passed as input to the scikit-learn implementation of MDS [1] called with maximum iterations set to 30,000 and number of initializations set to 10. For this analysis, HiCRep was run with the same settings as listed above.

Benchmarking For the timing and memory experiments, all three versions of HiCRep were run on a computer with Intel Xeon X5690 3.47 GHz CPUs and 48 GB of memory. For the results Our Python implementation of HiCRep was installed from PyPI and run with Python 3.7.6, NumPy 1.18.5, and SciPy 1.5.1, all of which were installed through the conda package manager. The R implementation was installed from conda's bioconda channel and run with R version 4.0.2, also installed from conda. In order to promote a fair comparison, we undertook measures to ensure that NumPy was not parallelizing any operations and that R was not forced to use swap space. The accession numbers of the five pairs of data sets used for the timing and memory experiments are 4DNFI9BA2KDU / 4DNFIAVRY6RG, 4DNFIEVNIWQ5 / 4DNFIBNTKRQT, 4DNFINIQYFKT / 4DNFIVCGZ44G, 4DNFIV3PIEQJ / 4DNFITPMHGXP, and 4DNFIOQLTI9G / 4DNFIH7MQHOR.

References

[1] F. Pedregosa, G. Varoquaux, A. Gramfort, V. Michel, B. Thirion, O. Grisel, M. Blondel, P. Prettenhofer, R. Weiss, V. Dubourg, J. Vanderplas, A. Passos, D. Cournapeau, M. Brucher, M. Perrot, and E. Duchesnay. Scikit-learn: Machine learning in Python. *Journal of Machine Learning Research*, 12:2825–2830, 2011.
